# On the use of sentiment analysis for linguistics research. Observations on sentiment polarity and the use of the progressive in Italian

**DOI:** 10.3389/frai.2023.1101364

**Published:** 2023-08-24

**Authors:** Lorella Viola

**Affiliations:** Luxembourg Centre for Contemporary and Digital History (C^2^DH), University of Luxembourg, Esch-sur-Alzette, Luxembourg

**Keywords:** sentiment (SEN) analysis, Italian, progressive form, subjectification, critical digital studies

## Abstract

This article offers a conceptual and methodological contribution to linguistics by exploring the potential value of using sentiment analysis (SA) for research in this field. Firstly, it discusses the limitations and advantages of using SA for linguistics research including the wider epistemological implications of its application outside of its original conception as a product reviews analysis tool. Methodologically, it tests its applicability against an established linguistic case: the correlation between subjective attitudes such as surprise, irritation and discontent and the use of the progressive. The language example is Italian for which this function of the progressive form has not been analyzed yet. The analysis applies FEEL-IT, a state-of-the-art transformer-based machine learning model for emotion and sentiment classification in Italian on language samples from various sources as collected in Evalita-2014 (238,556 words). The results show statistically significant correlations between negative subjective attitudes and the use of the progressive in line with previous accounts in other languages. The article concludes with a few additional propositions for practitioners and researchers using SA.

## Introduction

This article offers a conceptual and methodological contribution to linguistics by exploring the potential value of using sentiment analysis (SA) for research in this field. SA is a computational technique that allows the analyst to annotate language material for attitudes—either sentiment or opinions (Liu, 2020). It was first developed within natural language processing (NLP) studies as a sub-field of Information Retrieval (IR). Its original application was devised for inferring general opinions from online product reviews, a need that emerged from the explosion of e-commerce and social media of the early 2000's. As online product reviews started to overwhelm commercial companies with marketing material, previous methods such as surveys and focus groups quickly became obsolete; using SA was not only much faster but also infinitely cheaper.^1^ Since then, SA has been increasingly used in other domains of society such as in the health sector and by government agencies and for other tasks, including making stock market predictions and analyzing citizens' opinions or concerns (Liu, 2020). The aim of this paper is to investigate the limitations and advantages of the SA method for linguistics research, its applicability for tasks outside of its original conception, and to what extent it may be used in linguistics in which its use has mostly been adopted as a research method to enhance discourse analysis studies (Maite, 2016).

As a first distinctive feature, this study focuses on functional grammar (Dik, 1978, 1989), that is the study of grammar within its social, interactional, and cultural context (the so-called “functional paradigm”). Specifically, it tests the use of SA against an established case: the correlation between subjective attitudes such as surprise, irritation and discontent and the use of the progressive. Several accounts in various languages (e.g., English, German, and Dutch; Traugott and Dasher, 2001; Killie, 2004; Levin, 2013; Pfaff et al., 2013; Anthonissen et al., 2016, 2019) have indeed observed that non-aspectual pragmatic or subjective meanings, for example to signal politeness and/or discontent, may mark the use of progressive constructions. In this way, this article directly answers recent calls in linguistics for new analytical methods that could expand the field by devising novel and dynamic ways of exploring established topics (Rose and McKinley, 2020). However, with the exception of limited work in French (e.g., De Wit et al., 2020), studies have largely focused on Germanic languages and predominantly on English. As a second distinctive feature, here the use case is the Italian language. In this way, the study provides fresh insights into this line of enquiry by adding novel findings that could strengthen previous observations in a non-Germanic language.

As a third contribution, the article provides a conceptual analysis of SA as a research method used in domains outside of its original application and to answer research questions different from its original conception. It will be argued here that if on the one hand major technological advances, the explosion of data availability, and the increasingly mobile and multilingual world have called for new research designs, data collection techniques, and tools for analysis (e.g., Rose and McKinley, 2020), on the other such novel resources and methods require thorough understanding of the assumptions behind them, constant update, and critical supervision. For example, SA has been used in a variety of fields such as psychology (Salas-Zárate et al., 2017; Liu, 2022; Zhong and Ren, 2022), political science (Haselmayer and Jenny, 2017; Ansari et al., 2020; Matalon et al., 2021), social science (Karamibekr and Ghorbani, 2012; Bhat et al., 2020; Nguyen et al., 2020), digital humanities (Moreno-Ortiz, 2017; Moreno-Ortiz et al., 2020; Schmidt et al., 2021; Viola, 2022), and media studies (Burscher et al., 2016; Thelwall, 2016; Amarasekara and Grant, 2019) to analyze opinions about social and political issues, particularly on social media that is for tasks other than product review analysis. Hence, whilst recognizing the potential benefits of quantitative methods such as SA, the discussion of the limitations and advantages of using SA for linguistics research will contribute reflections on the wider epistemological implications of its application outside of product reviews analysis.

## Academic discussion

The explosion of digital material of the last two decades and the subsequent need to analyze it and interpret it paired with advances in technology and statistical theory have greatly impacted the way information is retrieved today (Viola, 2023). Disciplines across scientific areas have increasingly incorporated technology within their traditional workflows and developed sophisticated data-driven approaches to analyze ever larger and more complex datasets. As a result, computational methods such as SA are used more and more in domains outside of NLP and for tasks that are very different from their initial application (Drucker, 2020; Viola, 2022).

SA for example was originally conceived as a tool to maximize profits; through large-scale analyses of online product reviews, the goal behind the technique was to optimize marketing strategies.

Being SA first and foremost and economic instrument, several authors have pointed out how the application of this technique in domains different from its original conception and for tasks other than product reviews analysis poses several challenges, methodological but also epistemological (Pang and Lee, 2008; González-Bailón and Paltoglou, 2015; Puschmann and Powell, 2018; Viola, 2022, 2023). The main criticism addresses the critical issue that whereas SA will perform satisfactorily when rating opinions about products and services, opinions about social and political issues will likely be misclassified (Pang and Lee, 2008; Lee and Lau, 2020). This is because SA's algorithms lack sufficient background knowledge of the local social and political contexts, not to mention the much higher linguistic and cultural complexity of these types of texts compared to product reviews (e.g., sarcasm, puns, plays on words, and ironies; Liu, 2020). These scholars have therefore argued that this limitation makes the use of SA for empirical social research rather controversial, particularly when the method is borrowed uncritically by other disciplines or when the technique is embedded in a range of algorithmic decision-making systems (Karppi and Crawford, 2016; Puschmann and Powell, 2018; Viola, 2022, 2023).

Despite voices of skepticism around the technique, SA has shown no signs of decline over the years. To the contrary, computer science efforts have been increasingly devoted toward improving and refining the method, for instance by moving from dictionary-based approaches mostly context-agnostic to transformer-based machine learning models which go beyond single word predictions (see for instance Hassan and Mahmood, 2017; Shen et al., 2017; Meena et al., 2021, 2023; Ahmed et al., 2022; Li et al., 2022). Such continuing advances have certainly contributed to its growing adoption for empirical social research. More recently, for example, the application of SA to social media content has been used to analyze trends in public opinions and reactions to global concerns such as anxiety and stress in relation to diseases and health crises (e.g., Praveen et al., 2021; Jahanbin et al., 2022; Ogbuokiri et al., 2022; Meena et al., 2023).

This article discusses the gained prominence of SA within the wider context of two mutually reinforcing factors. First, the larger incorporation of technology in all sectors of society, naturally including also the creation of academic knowledge and second, the common misconception that computational outputs are objective and reliable, adding to the allure of the technique. It is argued here that such idealization of SA likely forms epistemological expectations that the method will inevitably disappoint. In their analysis of the perception of SA in public discourse, Puschmann and Powell (2018) for example highlight that the comforting illusion of objectivity and precision in relation to SA creates an expectation of validity and accuracy that is misaligned with the technique's original function (2), that is to only provide an approximation of human judgement (10).

Naturally, this misconception does not solely concern SA but all computational methods more widely as it pertains to the notion of discrete vs. continuous modeling of information (Calude and Longo, 2017; Longo, 2019; Viola, 2023). In discrete systems, information is rendered as exact and separate points—sequences of 0 and 1s—and something belongs to either one category or another. For example, a SA task is usually modeled as a classification problem, that is a classifier processes pre-defined elements in a text (e.g., sentences) and it returns a pre-set category (e.g., positive, negative, or neutral). The resulted discrete output produces an illusion of accuracy; it is argued here however that it is the process itself of discretizing emotions that poses several challenges, including the assumption that it is possible to not only disambiguate subjectivity, but also to quantify attitudes and even attribute them scores.

Over the years computer scientists have attempted to develop more sophisticated SA methods that aimed to alleviate issues such as reducing the subjective perception of emotions to two/three (unproblematized) categories and identifying the multiple elements that different types of sentiment may refer to in the same text. For example, through so-called “fine-grained classifiers,” the algorithm extracts information to discriminate aspects and opinion targets (Schouten and Frasincar, 2015; Pontiki et al., 2016) and provides a slightly less rigid classification output (e.g., very positive, positive, neutral, negative, and very negative) thus adding a more nuanced distinction of the identified sentiment (e.g., Munikar et al., 2019). Other classifiers return a prediction of the corresponding sentiment (e.g., anger, happiness, and sadness; e.g., Kawade and Oza, 2017); others yet try to quantify how much emotional content is present within the document (i.e., sentiment magnitude; e.g., Jini and Prabu, 2019). To overcome the issue of being context agnostic, attempts have also been made to incorporate external linguistic content such as historical data into the model (Xiao et al., 2022).

From a linguistic and epistemological point of view, however, it is the intrinsic conceptualization of emotions as quantifiable, discrete, fixed, and objective entities that raises doubts about the legitimacy of the technique, particularly for empirical social research. Viola (2023, p. 72) argues:

[…] we are told that SA is a quantitative method that provides us with a picture of opinionated trends in large amounts of material otherwise impossible to map. In reality, the reduction of something as idiosyncratic as the definition of human emotions to two/three categories is highly problematic as it hides the whole set of assumptions behind the very establishment of such categories. For example, it remains unclear what is meant by neutral, positive, or negative as these labels are typically presented as a given, as if these were unambiguous categories universally accepted (Puschmann and Powell, 2018—quoted in the original).

Indeed, the classification of linguistic categories is a well-known linguistic problem. Langacker (1983) stated that the subjective, processual, and context-bound nature of language in use prevents linguistic categories from being unequivocally defined (see also Talmy, 2000; Croft and Cruse, 2004; Dancygier and Sweetser, 2012; Gärdenfors, 2014; Paradis, 2015). In challenging tasks such as manually annotating language material, this issue becomes particularly apparent. Indeed, when several human annotators are asked to annotate language material, there is always an expectation of disagreement on same annotation decisions. This expectation is known as “inter-annotator agreement,” a measure that calculates the degree of agreement between the annotators' decisions about a label and is meant to function as a warning to the analyst before drawing any linguistic conclusion uniquely based on manually annotated language material. The inter-annotator agreement measure may vary greatly as it factors several parameters into the calculation (e.g., number of annotators, number of categories, and type of text) but in general, it is never expected to be 100%. Especially when the annotation concerns highly subjective linguistic elements whose interpretation is inseparable from the annotators' culture, personal experiences, values, and beliefs—such as the perception of sentiment—this percentage has been found to remain at 60–65% at best (Bobicev and Sokolova, 2017, 2018).

Consequently, it should further be noted that any conclusion based on SA output will inevitably include an additional degree of inconsistency between the way the categories of positive, negative, and neutral emotion have been assigned in the model and the analyzed material to which the model is applied. For this reason, applying a SA model across different textual genres is not advisable as the sentiment will likely be misclassified.

The academic discussion has highlighted the complexities and ambiguities of SA as a method for empirical social research. Particularly when used for tasks other than its original design, this article argues that the positivist hypes brought about by the digital transformation of society and the increasing incorporation of computational methods into academic knowledge production should not obfuscate the promise of SA, that is to function as an approximation of—and not a substitute for—human judgement. It is within this academic discussion that the article tests the potential value of using SA in linguistics; in doing so, it attempts to answer the following research questions: (1) to what extent is SA a suitable method for linguistics research? To answer this question, the article applies SA to an existing case in functional grammar, the subjectification of the progressive form. This leads to the second research question: (2) can SA be used to find correlations between subjective attitudes and the use of the progressive in Italian?

## Subjectification and the progressive form

The study of the progressive construction has fascinated linguists for a long time; this is probably due to the various functions it performs as well as its continuing changing nature. As it escapes a single-level taxonomy, over the years several mappings have been suggested across languages, mostly based on the progressive's internal characteristics of aspectuality, imperfectivity, and incompleteness. More recently, however, the progressive has started to be explored through the lens of pragmatics and discourse; these observations suggest that this chameleonic form can also function as a marker of non-aspectual pragmatic or subjective meanings, for example to signal surprise, politeness, irritation, and discontent (Traugott and Dasher, 2001; Killie, 2004; Levin, 2013; Pfaff et al., 2013; Anthonissen et al., 2016, 2019; Freund, 2016; Martínez-Vázquez, 2018). In other words, alongside other linguistic structures such as epistemic modality (e.g., *He could have done better*; Traugott, 1989, 1995) and English sentence adverbs (e.g., *Clearly, you know what you're doing*; Swan and Breivik, 1997, 2011), progressive constructions may also be examples of subjectification, defined as the “semantic-pragmatic process whereby ‘meanings become increasingly based in the speaker's subjective belief state/attitude toward the proposition,' in other words, toward what the speaker is talking about” (Traugott, 1995, p. 31).

Authors have advanced various hypotheses that may explain this phenomenon. Scheffer (1975) for example suggests that in English, the progressive may be used subjectively by the speaker with reference to the temporal aspect of the verb, for example to emphasize the excessive duration of an event and therefore to manifest irritation. In addition to this aspect of temporality, Bland (1988) and Smitterberg (2005) also argue that the English progressive may equally signal subjectivity when the intention is to emphasize the intensity of an emotion or a situation (e.g., *I'm loving this*). Other accounts in French also show similar results. In their analysis of modality and aspect in the progressive, De Wit and Patard (2013) for example found that the progressive can also be used to signal irritation and surprise but also to express hedge in reference to an event. More recently, in a comparative study on language samples in English, Dutch and French, De Wit et al. (2020) argued that present progressive constructions are particularly liable to be used for signaling that something about the ongoing situation is unconventional, what they call “extravagant language.”

Although findings on the use of the progressive to mark subjective meanings have been validated independently by several studies on different datasets, the majority of these observations come from Germanic languages and besides the few already mentioned exceptions in French, this function has not been explored in other Roman languages yet. By investigating the subjective dimension of the progressive in a typologically diverse language like Italian, this study aims to contribute novel findings to this area of investigation, expanding the field and contributing new details to existing theories and methods.

## Methodology and dataset

This article investigates the applicability of SA as an experimental method for linguistics research. As a methodological and conceptual contribution, the article aims to explore new analytical paradigms and tools that may help linguists to meet the challenge of analyzing ever larger and more complex language material. For the analysis, the study uses FEEL-IT (Bianchi et al., 2021), a state-of-the-art transformer-based machine learning classifier for emotion and sentiment classification in Italian to predict sentiments and identify the corresponding emotions. It employs the Italian BERT model UmBERTo trained on Commoncrawl ITA (Parisi et al., 2021) and fine-tuned on a corpus by the same name containing Italian Twitter posts manually annotated with four basic emotions: anger, fear, joy, sadness. This SA classifier returns a prediction of the sentiment polarity (i.e., positive or negative) and the specific corresponding sentiment (i.e., anger, joy, sadness, fear). The sentiment polarity is obtained by collapsing the four emotions into the two categories (i.e., joy → positive; fear, anger, sadness → negative). Table 1 displays the results of emotion recognition models trained on FEEL-IT and tested on two other datasets: MultiEmotions-It (ME; Sprugnoli, 2021) and a dataset of 662 tweets about COVID-19 (C-19) against the Most Frequent Class (MFC) for baseline results. As the table shows, the model is stable and accuracy is acceptable.

**Table 1:** Results of emotion recognition models trained on FEEL-IT.

**Model**	**Testing**	**P**	**R**	**F1**	**Acc**
UmBERT0-FT	ME	0.56	0.59	0.57	0.73
UmBERT0-PT	ME	0.56	0.66	0.57	0.69
MFC	ME	0.16	0.25	0.20	0.64
UmBERT0-FT	C19	0.56	0.56	0.56	0.69
UmBERT0-PT	C19	0.53	0.53	0.50	0.60
MFC	C19	0.15	0.25	0.19	0.60

Precision (P), Recall (R), F1-score (F1), and Accuracy (Acc) of emotion recognition on use-cases using UmBERTo model (Bianchi et al., 2021).

Another challenge of using computational methods for linguistic research is related to the predominance of models, datasets, and tools devised and developed for the English language. Even when models and resources in other languages exist, they are often proprietary and expensive (e.g., Google Cloud Platform Console) as well as not transparent often offering only opaque documentation. Because not all languages are equally resourced digitally and computationally, researchers, teachers and curators are forced to compromise on which tasks can be performed, with which tools and through which platforms (Viola, 2023). Such Anglophone-centricity is therefore often still a barrier for researchers working with languages other than English (Viola and Fiscarelli, 2021; Viola, 2023; Viola et al., 2023). Resources like FEEL-IT are important tools to counterbalance the predominance of English in computer science.

The dataset for the analysis is the Italian language data used in the EVALITA Parsing Task project.^2^ This is a periodic evaluation campaign of Natural Language Processing (NLP) and speech tools for the Italian language. The aim of the EVALITA project is to promote the development of language and speech technologies for the Italian language and to offer to the scientific community a shared framework where different systems and approaches can be evaluated consistently. The corpus used here is the training and test data corpora used for the 2014 EVALITA task. It contains language samples from various sources (e.g., legal texts, news articles, and Wikipedia articles) collected from 2011 to 2014 and it totals up to 238,556 words.

After launching the SA model on the dataset, random excerpts are analyzed qualitatively to assess whether the assigned prediction can be reputed reliable. Specifically, the analysis uses the language data provided by ISDT (Italian Stanford Dependency Treebank) released for the dependency parsing shared task of Evalita-2014 (Bosco et al., 2014).

The analysis proceeds at two levels, quantitative and qualitative. First, the quantitative analysis aims to find statistically significant correlations between sentiment polarity and the use of the progressive; second, the qualitative analysis aims to assess to what extent the sentiment and emotion classification can be reputed reliable. In the first stage, the language material will first be tokenized into sentences and filtered for verbs at the progressive form. This will allow us to work on a subset containing only the relevant verbal constructions thus making the SA processing faster and more efficient. The SA output will then be tested for significance. In the second phase, random excerpts will be qualitatively analyzed against the classified emotion and polarity.

## Analysis and results

**Figure 1:**
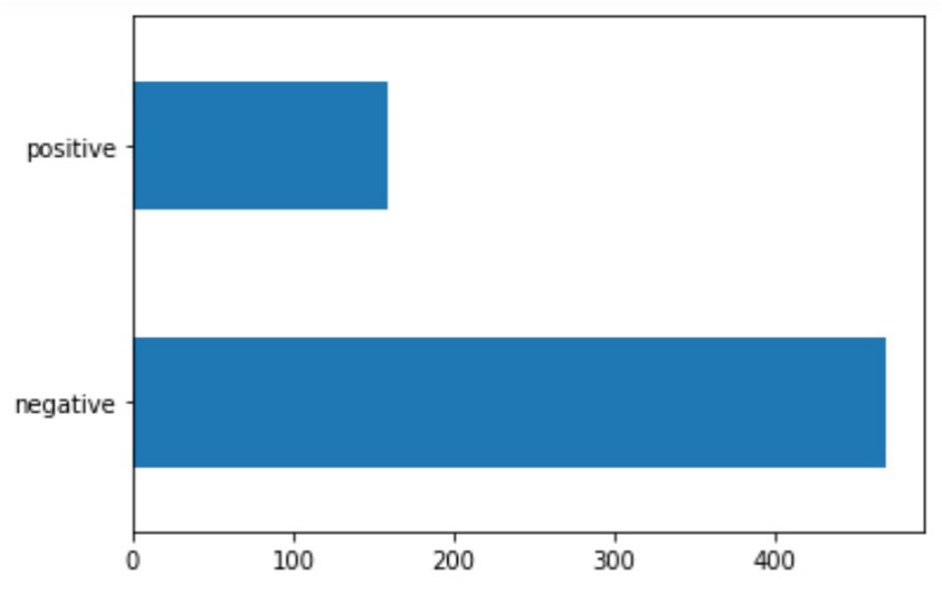
Distribution of sentiment in the subset corpus.

**Figure 2:**
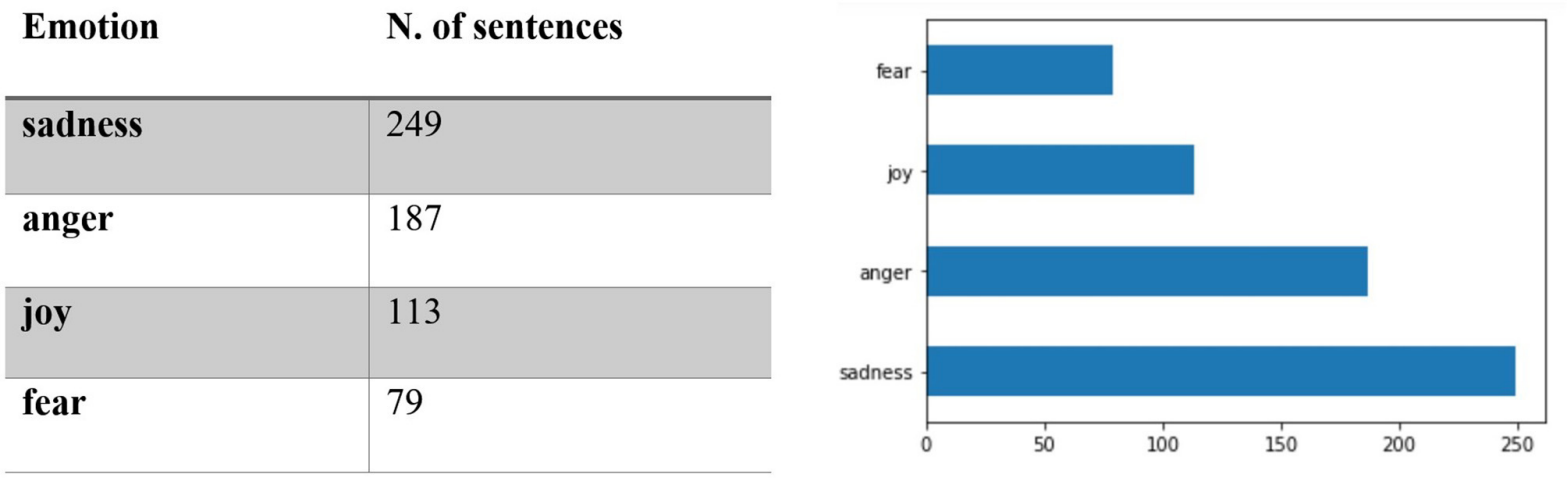
Distribution of the identified emotion in the subset corpus.

The relevant subset contains 628 sentences containing 377 verbs at the gerund/progressive form of which 382 from the first declination (verbs ending in *-are*) and 248 from the second declination (verbs ending in –*ere*; none from the third). The subset was then queried for sentiment using the FEEL-IT Python library. The results showed that the majority of the sentences in the subset (469 vs. 159) were classified as negative (Figure 1); Figure 2 reports the distribution of the identified corresponding emotion.

**Figure 3:**
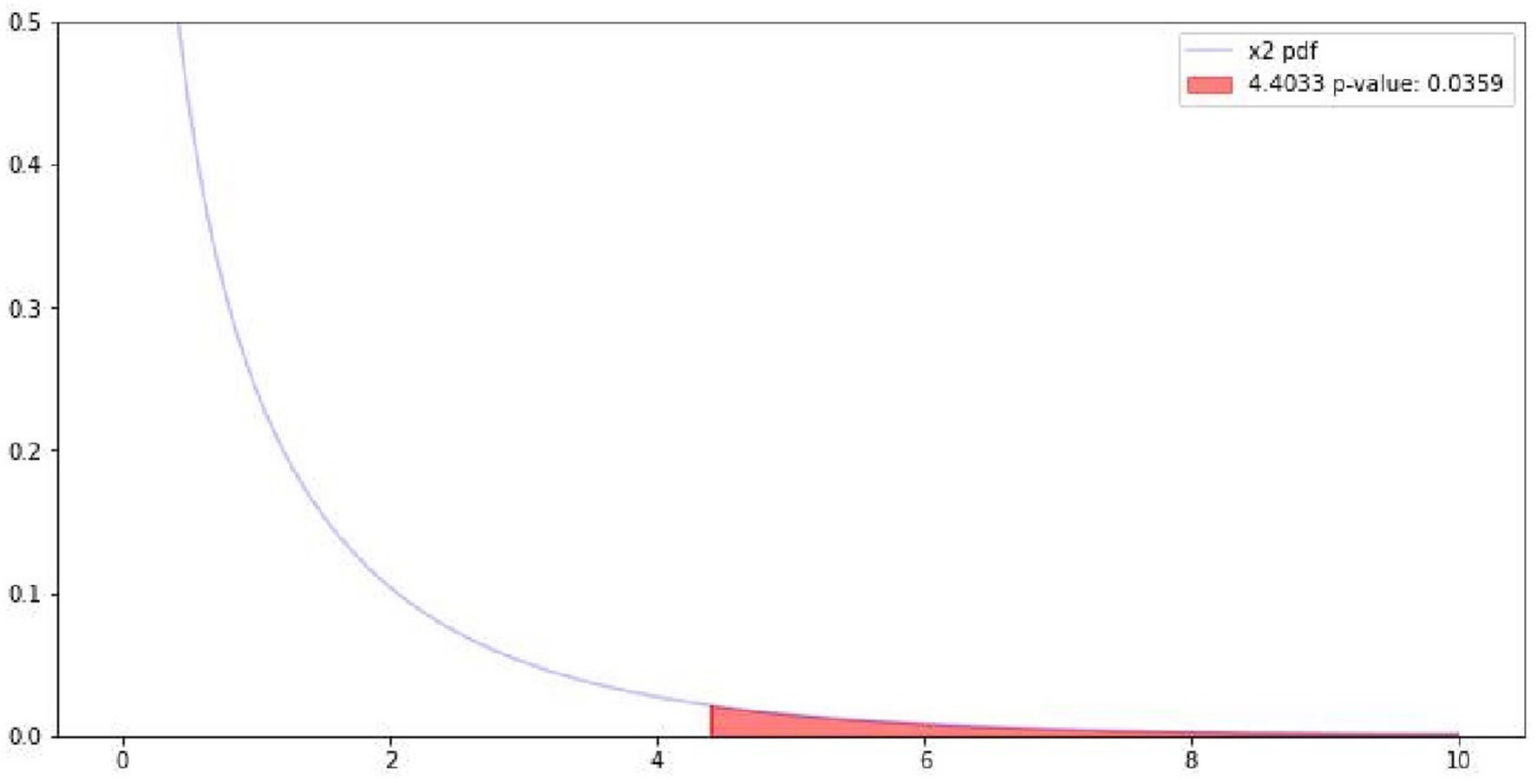
Visualization of Chi-square results. The red section represents the p-value.

Following the quantitative analysis, the results were tested for significance; Table 2 reports the results of the chi-square test (also displayed in Figure 3).

**Table 2:** Chi-square results for the correlation between gerund/progressive forms and sentiment polarity.

	**Chi-square test**	**Results**
0	Pearson Chi-square (1.0) =	4.4033
1	*p*-value =	0.0359
2	Cramer's phi =	0.0182

The Chi-square results align with previous findings in other languages showing that statistically significant correlations between negative sentiment and the use of gerund/progressive constructions can be observed. However, even though the correlation is significant, Cramer's phi reports that the strength of the relationship is not high. This finding could be explained by several factors including the diversity of the sources in the dataset, the different genres, the fact that SA is applied to short sentences and therefore that very limited context is provided. Future investigations could for example perform logistic regression to identify other potential factors that are predictive of sentiment (e.g., type of sources, type of verb, and topics).

### Qualitative analysis

This section presents the qualitative analysis of random excerpts^3^; the aim is to assess to what extent the sentiment and emotion classification can be reputed reliable (bold added).

#### Excerpt 1

*La verita' e' che ormai la spesa sanitaria **sta esplodendo** per motivi che non hanno nulla a che vedere con il miglioramento del servizio ed e' un incremento che neppure gli assessori sanno spiegare*.

*The truth is that now health costs* ***are exploding** for reasons that have nothing to do with service improvements and that not even politicians can explain*.

The sentence in (1) was classified as negative and the corresponding emotion was classified as anger. Although the identified emotion may be correct to an extent, the attributed^3^ classification (i.e., negative) may not be aligned with the writer's original intentions. Indeed, one often unclear element of SA is the clarification of whether the algorithm detects the attitude of the writer or the expressed polarity in the analyzed textual fragment (Puschmann and Powell, 2018; Viola, 2023). This is a known limitation of classification systems such as SA that are based on rigid categories (e.g., positive/negative, anger = negative). If taken individually the issue may be negligible, in the aggregate misclassifications like this one are amplified by the scale of the analyzed material, which can run in the order of millions of sentences. It is true that thanks to the additional provision of the corresponding emotion, more nuanced systems like FEEL-IT partially overcome this limitation; analysts and researchers should however carefully assess to what extent they should rely on the SA's output and always consider it as an approximation of human judgement.

#### Excerpt 2

*I partiti d'opposizione al Governo di destra del presidente Berisha* ***stanno*** ***soffiando** sul fuoco delle proteste e hanno indetto una manifestazione per oggi nella capitale*.

*Left-wing opposition parties of President Berisha's Government* ***are blowing*** ***over** the fire of the protests and have announced a march for today in the capital*.

The sentence in (2) was classified as negative and the corresponding emotion was classified as anger. At the time when this material was collected (2011–2014), President Sali Berisha was Prime Minister of Albania. His government was the center of long controversies and protests by the socialist party which accused him of power abuse, corruption and human rights violation. Once again, the emotion seems to have been correctly classified but because in the model, *anger* has been collapsed into negative sentiment polarity, regardless of the context the sentence is classified as negative. Again, analysts using SA, particularly for empirical social science research, should be aware that there is always a degree of inconsistency between the way the categories of positive/negative have been defined in the training model and the writer's intention in the actual material to which the model is applied.

## Discussion

The results showed statistically significant correlations between negative subjective attitudes such as anger and sadness and the use of the progressive, in line with previous accounts in English, German, French, and Dutch. A close inspection of random excerpts highlighted some of the uncertainties of using this technique in fields other than IR and for tasks other than product review analysis, such as for empirical social research. For example, it was found a potential degree of discrepancy between the writer's intention, the way the classification was carried out for training the SA model, and the text to which the model was applied. The issue is related to the reduction of opinions and sentiment to two/three categories, conceptually problematic when applied to social data. At the same time, the provision of a more nuanced classification system such as the prediction of the corresponding emotion may contribute to alleviate this issue. It is therefore recommended that analysts prefer finer-grained classifiers over those providing solely a basic identification of sentiment, as this may introduce errors and biases in the final output.

It is argued here that these uncertainties add further complexity and ambiguity to the already existing limitations of the technique and that such complexities and limitations should be assessed carefully by researchers and practitioners before and during the analysis.

## Future studies

Whereas the correlation between negative sentiment and the progressive was found statistically significant, the strength of the relationship was not high. Future research could perform regression analysis to explore other predictors of sentiment polarity in connection with the progressive. Moreover, this line of enquiry would benefit from further experiments using manually annotated Italian data for sentiment analysis, emotion identification/recognition tasks. Finally, larger datasets could be used in future research, including in other languages where this relationship has already been attested (i.e., English, German, Dutch, and French). This could either further validate the suitability of SA for this line of research or discard it.

## Conclusion

This article provided a conceptual and methodological contribution to linguistics by investigating the applicability of SA for research in this field with a focus on functional grammar. The aim was to add to the scholarly conversation by testing the potential value of this computational method to navigate the complexities of large datasets of language material. In doing so, the study answered to recent calls in the linguistics field for incorporating more advanced techniques than traditional Corpus Linguistics approaches. The analysis tested the applicability of SA against an established linguistic case—the subjective use of the progressive form—and it took Italian as a case study. The language choice was motivated by the fact that while the subjective function of the progressive has been observed in Germanic languages and in French, there are no study empirically investigating this function in Italian. Thus, the results of this study contributed fresh findings to this body of work.

The article also discussed the limitations and advantages of using SA for linguistics research and for empirical social research more widely and examined specifically the larger epistemological implications of applying this method for tasks outside of its original conception. As language repositories become ever larger and social media texts become more and more the preferred lens through which social scientists and linguists conduct their investigations, traditional quantitative approaches may not fully capture the complexities of digital communication material such as those brought about by the increasing volume of available data. Traditional methods alone, including for example words' collocation analyses, may no longer be sufficient for identifying otherwise not immediately evident patterns and discontinuities. More sophisticated methods such as SA can therefore be of great assistance to linguists who are now increasingly confronted with the challenge of analyzing the complexities of the textual material produced and fairly easily available. However, although undoubtedly still providing powerful means to navigate large quantities of language material, these methods should not be adopted uncritically. Researchers and practitioners using SA should therefore use this technique as an exploratory method, particularly when applied to empirical social research and always resize their epistemological expectations accordingly. At the very least, an acknowledgment of such complexities should be present when using this technique.

## Data availability statement

Publicly available datasets were analyzed in this study. This data can be found at: Evalita.it.

## Author contributions

The author confirms being the sole contributor of this work and has approved it for publication.

## Footnotes

^1^For a recent overview of sentiment analysis please see Lee and Lau (2020).

^2^ https://www.evalita.it/

^3^Translations by the author.

## References

[B1] Ahmed K. Nadeem M. I. Li D. Zheng Z. Ghadi Y. Y. Assam M.. (2022). Exploiting stacked autoencoders for improved sentiment analysis. Appl. Sci. 12, 12380. 10.3390/app122312380

[B2] Amarasekara I. Grant W. J. (2019). Exploring the YouTube science communication gender gap: a sentiment analysis. Publ. Understand. Sci. 28, 68–84. 10.1177/0963662518786654 29974815

[B3] Ansari M. Z. Aziz M. B. Siddiqui M. O. Mehra H. Singh K. P. (2020). Analysis of political sentiment orientations on Twitter. Proc. Comput. Sci. 167, 1821–1828. 10.1016/j.procs.2020.03.201

[B4] Anthonissen L. De Wit A. Mortelmans T. (2016). Aspect meets modality: a semantic analysis of the German Am -Progressive. J. Germanic Linguist. 28, 1–30. 10.1017/S1470542715000185

[B5] Anthonissen L. De Wit A. Mortelmans T. (2019). (Inter)subjective uses of the dutch progressive constructions. Linguistics 57, 1111–1159. 10.1515/ling-2019-0019

[B6] Bhat M. Qadri M. Beg N. A. Kundroo M. Ahanger N. Agarwal B. (2020). Sentiment analysis of social media response on the COVID-19 outbreak. Brain Behav. Immunity 87, 136–137. 10.1016/j.bbi.2020.05.006 32418721 PMC7207131

[B7] Bianchi F. Nozza D. Hovy D. (2021). “FEEL-IT: emotion and sentiment classification for the Italian language,” in Proceedings of the Eleventh Workshop on Computational Approaches to Subjectivity, Sentiment and Social Media Analysis (Online: Association for Computational Linguistic), 76–83. Available online at: https://www.aclweb.org/anthology/2021.wassa-1.8 (accessed August 16, 2023).

[B8] Bland S. K. (1988). The present progressive in discourse: grammar versus usage revisited. TESOL Quart. 22, 53. 10.2307/3587061

[B9] Bobicev V. Sokolova M. (2017). “Inter-annotator agreement in sentiment analysis: machine learning perspective,” in RANLP 2017—Recent Advances in Natural Language Processing Meet Deep Learning (Shoumen: Incoma Ltd.), 97–102. 10.26615/978-954-452-049-6_015

[B10] Bobicev V. Sokolova M. (2018). Thumbs up and down: sentiment analysis of medical online forums. EMNLP 2018, 5906. 10.18653/v1/W18-5906

[B11] Bosco C. Dell'Orletta F. Montemagni S. Sanguinetti M. Simi M. (2014). “The EVALITA 2014 dependency parsing task,” in EVALITA 2014 Evaluation of NLP and Speech Tools for Italian (Pisa: Pisa University Press), 1–8. 10.12871/clicit201421

[B12] Burscher B. Vliegenthart R. de Vreese C. H. (2016). Frames beyond words: applying cluster and sentiment analysis to news coverage of the nuclear power issue. Soc. Sci. Comput. Rev. 34, 530–545. 10.1177/0894439315596385

[B13] Calude C. S. Longo G. (2017). The deluge of spurious correlations in big data. Found. Sci. 22, 595–612. 10.1007/s10699-016-9489-4

[B14] Croft W. Cruse D. A. (2004). Cognitive Linguistics, 1st Edn. Cambridge University Press. 10.1017/CBO9780511803864

[B15] Dancygier B. Sweetser E. (2012). Viewpoint in Language: A Multimodal Perspective. Cambridge: Cambridge University Press. 10.1017/CBO9781139084727

[B16] De Wit A. Patard A. (2013). Modality, aspect and the progressive: the semantics of the present progressive in french in comparison with english. Lang. Contrast 13, 113–132. 10.1075/lic.13.1.06wit

[B17] De Wit A. Petré P. Brisard F. (2020). Standing out with the progressive. J. Linguist. 56, 479–514. 10.1017/S0022226719000501

[B18] Dik S. C. (1978). Functional Grammar. 3rd Edn. Berlin; Boston, MA: De Gruyter.

[B19] Dik S. C. (1989). The Theory of Functional Grammar: The Structure of the Clause. Berlin: Walter de Gruyter.

[B20] Drucker J. (2020). Visualization and Interpretation: Humanistic Approaches to Display. Cambridge, MA: The MIT Press.

[B21] Freund N. (2016). Recent change in the use of stative verbs in the progressive form in British English : i'm loving it. Lang. Stud. Work. Pap. 7, 50–61.

[B22] Gärdenfors P. (2014). The Geometry of Meaning: Semantics Based on Conceptual Spaces. Cambridge, MA: MIT Press.

[B23] González-Bailón S. Paltoglou G. (2015). Signals of public opinion in online communication: a comparison of methods and data sources. Annal. Am. Acad. Polit. Soc. Sci. 659, 95–107. 10.1177/0002716215569192

[B24] Haselmayer M. Jenny M. (2017). Sentiment analysis of political communication: combining a dictionary approach with crowdcoding. Qual. Quant. 51, 2623–2646. 10.1007/s11135-016-0412-4 29070915 PMC5635074

[B25] Hassan A. Mahmood A. (2017). “Deep learning approach for sentiment analysis of short texts,” in 2017 3rd International Conference on Control, Automation and Robotics (ICCAR) (Nagoya), 705–710. 10.1109/ICCAR.2017.7942788

[B26] Jahanbin K. Jokar M. Rahmanian V. (2022). Using twitter and web news mining to predict the monkeypox outbreak. Asian Pacific J. Trop. Med. 15, 236. 10.4103/1995-7645.346083

[B27] Jini J. S. Prabu P. (2019). Detecting the magnitude of depression in twitter users using sentiment analysis. Int. J. Electr. Comput. Eng. 9, 3247. 10.11591/ijece.v9i4.pp3247-3255

[B28] Karamibekr M. Ghorbani A. A. (2012). “Sentiment analysis of social issues.” in 2012 International Conference on Social Informatics (Alexandria, VA), 215–221. 10.1109/SocialInformatics.2012.49

[B29] Karppi T. Crawford K. (2016). Social media, financial algorithms and the hack crash. Theory Cult. Soc. 33, 73–92. 10.1177/0263276415583139

[B30] Kawade D. R. Oza K. S. (2017). Sentiment analysis: machine learning approach. Int. J. Eng. Technol. 9, 2183–2186. 10.21817/ijet/2017/v9i3/170903015136789379

[B31] Killie K. (2004). Subjectivity and the English progressive. Engl. Lang. Linguist. 8, 25–46. 10.1017/S1360674304001236

[B32] Langacker R. W. (1983). Foundations of Cognitive Grammar. Bloomington, IN: Indiana University Linguistics Club.

[B33] Lee S. Y. M. Lau H. Y. P. (2020). “An event-comment social media corpus for implicit emotion analysis,” in Proceedings of The 12th Language Resources and Evaluation Conference (Marseille: European Language Resources Association), 1633–1642. Available online at: https://www.aclweb.org/anthology/2020.lrec-1.203 (accessed August 16, 2023).

[B34] Levin M. (2013). “The progressive verb in modern American English,” in The Verb Phrase in English: Investigating Recent Language Change with Corpora, eds Bas, A., G. Leech, J. Close, and S. Wallis (Cambridge: Cambridge University Press), 187–216. 10.1017/CBO9781139060998.009

[B35] Li D. Ahmed K. Zheng Z. Mohsan S. A. H. Alsharif M. H. Hadjouni M.. (2022). Roman urdu sentiment analysis using transfer learning. Appl. Sci. 12, 10344. 10.1007/978-3-031-12762-5

[B36] Liu B. (2020). Sentiment Analysis: Mining Opinions, Sentiments, and Emotions. Cambridge: Cambridge University Press.

[B37] Liu X. (2022). Analysis of psychological characteristics and emotional expression based on deep learning in higher vocational music education. Front. Psychol. 13, 981738. 10.3389/fpsyg.2022.981738 36211911 PMC9537096

[B38] Longo G. (2019). Quantifying the world and its webs: mathematical discrete vs. continua in knowledge construction. Theory Cult. Soc. 36, 63–72. 10.1177/0263276419840414

[B39] Maite T. (2016). Sentiment Analysis: An Overview from Linguistics. Annual Review of Lingui. 2:325–47. 10.1146/annurev-linguistics-011415-040518

[B40] Martínez-Vázquez M. (2018). I'm loving it! A corpus-based study of the progress of love. J. Engl. Linguist. 46, 140–166. 10.1177/0075424218765609

[B41] Matalon Y. Magdaci O. Almozlino A. Yamin D. (2021). Using sentiment analysis to predict opinion inversion in tweets of political communication. Sci. Rep. 11, 7250. 10.1038/s41598-021-86510-w 33790339 PMC8012385

[B42] Meena G. Mohbey K. K. Indian A. (2021). Categorizing sentiment polarities in social networks data using convolutional neural network. SN Comput. Sci. 3, 116. 10.1007/s42979-021-00993-y

[B43] Meena G. Mohbey K. K. Kumar S. Lokesh K. (2023). A hybrid deep learning approach for detecting sentiment polarities and knowledge graph representation on monkeypox tweets. Decision Analyt. J. 7, 100243. 10.1016/j.dajour.2023.100243

[B44] Moreno-Ortiz A. (2017). “Lingmotif: sentiment analysis for the digital humanities,” in Proceedings of the Software Demonstrations of the 15th Conference of the European Chapter of the Association for Computational Linguistics (Valencia: Association for Computational Linguistics), 73–76. Available online at: https://aclanthology.org/E17-3019 (accessed August 16, 2023).

[B45] Moreno-Ortiz A. Fernandez-Cruz J. Hernández C. P. C. (2020). “Design and evaluation of SentiEcon: a fine-grained economic/financial sentiment lexicon from a corpus of business news,” in Proceedings of The 12th Language Resources and Evaluation Conference (Marseille: European Language Resources Association), 5065–5072. Available online at: https://www.aclweb.org/anthology/2020.lrec-1.623 (accessed August 16, 2023).

[B46] Munikar M. Shakya S. Shrestha A. (2019). Fine-grained sentiment classification using BERT. arXiv. 10.1109/AITB48515.2019.8947435

[B47] Nguyen D. Liakata M. DeDeo S. Eisenstein J. Mimno D. Tromble R.. (2020). How we do things with words: analyzing text as social and cultural data. Front. Artif. Intell. 3, 62. 10.3389/frai.2020.00062 33733179 PMC7861331

[B48] Ogbuokiri B. Ahmadi A. Nia Z. M. Mellado B. Wu J. Orbinski J.. (2022). Vaccine hesitancy hotspots in Africa: an insight from geotagged twitter posts. TechRxiv. 10.36227/techrxiv.20720740.v1

[B49] Pang B. Lee L. (2008). Opinion mining and sentiment analysis. Found. Trends^®^ Inform. Retrieval 2, 1–135. 10.1561/9781601981516

[B50] Paradis C. (2015). “Conceptual spaces at work in sensory cognition: Domains, dimensions and distances,” in Applications of Conceptual Spaces (Springer), 33–55.

[B51] Parisi L. Francia S. Magnani P. (2021). Musixmatchresearch/Umberto. Python. Musixmatch Research. Available online at: https://github.com/musixmatchresearch/umberto (accessed August 16, 2023).

[B52] Pfaff M. Bergs A. Hoffmann T. (2013). “I was just reading this article– on the expression of recentness and the English past progressive,” in The Verb Phrase in English: Investigating Recent Language Change with Corpora, eds Bas, A., G. Leech, J. Close, and S. Wallis (Cambridge: Cambridge University Press), 217–238. 10.1017/CBO9781139060998.010

[B53] Pontiki M. Galanis D. Papageorgiou H. Androutsopoulos I. Manandhar S. Al-Smadi M.. (2016). “Semeval-2016 task 5, aspect based sentiment analysis,” in ProWorkshop on Semantic Evaluation (SemEval-2016), eds S. Bethard, M. Carpuat, D. Cer, D. Jurgens, P. Nakov, and T. Zesch (San Diego, CA: Association for Computational Linguistics), 19–30. 10.18653/v1/S16-1002

[B54] Praveen S. V. Ittamalla R. Deepak G. (2021). Analyzing Indian general public's perspective on anxiety, stress and trauma during COVID-19—A machine learning study of 840,000 tweets. Diabet. Metabol. Syndr. 15, 667–671. 10.1016/j.dsx.2021.03.016 33813239

[B55] Puschmann C. Powell A. (2018). Turning words into consumer preferences: how sentiment analysis is framed in research and the news media. Soc. Media Soc. 4, 205630511879772. 10.1177/2056305118797724

[B56] Rose H. McKinley J. (2020). The Routledge Handbook of Research Methods in Applied Linguistics. Routledge Handbooks in Applied Linguistics. New York, NT: Routledge.

[B57] Salas-Zárate M. P. Pilar M. Paredes-Valverde M. A. Rodríguez-García M. Á. Valencia-García R. Alor-Hernández G. (2017). “Sentiment analysis based on psychological and linguistic features for Spanish language,” in Current Trends on Knowledge-Based Systems, eds G. Alor-Hernández and R. Valencia-García (Intelligent Systems Reference Library. Cham: Springer International Publishing), 73–92. 10.1007/978-3-319-51905-0_4

[B58] Scheffer J. (1975). The Progressive in English. Amsterdam: North-Holland Pub.Co.; New York, NY: American Elsevier Publishing Company. Available online at: http://archive.org/details/progressiveineng0000sche (accessed August 16, 2023).

[B59] Schmidt T. Dangel J. Wolff C. (2021). SentText: A Tool for Lexicon-Based Sentiment Analysis in Digital Humanities. Regensburg: Universität Regensburg.

[B60] Schouten K. Frasincar F. (2015). Survey on aspect-level sentiment analysis. IEEE Trans. Knowl. Data Eng. 28, 813–830. 10.1109/TKDE.2015.2485209

[B61] Shen Q. Wang Z. Sun Y. (2017). “Sentiment analysis of movie reviews based on CNN-BLSTM,” in Intelligence Science I, eds Z. Shi, B. Goertzel, and J. Feng (IFIP Advances in Information and Communication Technology; Cham: Springer International Publishing), 164–171. 10.1007/978-3-319-68121-4_17

[B62] Smitterberg E. (2005). The Progressive in 19th-Century English: A Process of Integration. Language and Computers, N° *54*. Amsterdam: Rodopi.

[B63] Sprugnoli R. (2021). “MultiEmotions-It: a new dataset for opinion polarity and emotion analysis for Italian,” in Proceedings of the Seventh Italian Conference on Computational Linguistics, Vol. 2769. CEUR Workshop Proceedings, eds J. Monti, F. Dell'Orletta, and F. Tamburini (Bologna: CEUR). Available online at: https://ceur-ws.org/Vol-2769/#8 (accessed August 16, 2023).

[B64] Swan T. Breivik L. E. (1997). “Subject-oriented adverbs in a diachronic and contrastive perspective,” in Language History and Linguistic Modelling. Trends in Linguistics. Studies and Monographs, eds R. Hickey and S. Puppel. (Berlin; New York, NY: De Gruyter Mouton), 395–422. 10.1515/9783110820751.395

[B65] Swan T. Breivik L. E. (2011). English sentence adverbials in a discourse and cognitive perspective. Engl. Stud. 92, 679–692. 10.1080/0013838X.2011.604917

[B66] Talmy L. (2000). Toward a Cognitive Semantics. MIT Press.

[B67] Thelwall M. (2016). “Sentiment analysis,” in The SAGE Handbook of Social Media Research Methods, eds L. Sloan and A. Quan-Haase (London: SAGE Publications Ltd), 545–556. 10.4135/9781473983847.n32

[B68] Traugott E. C. (1989). On the rise of epistemic meanings in english: an example of subjectification in semantic change. Language 65, 31–55. 10.2307/414841

[B69] Traugott E. C. (1995). “Subjectification in grammaticalisation,” in Subjectivity and Subjectivisation: Linguistic Perspectives, eds D. Stein and S. Wright (Cambridge: Cambridge University Press), 31–54. 10.1017/CBO9780511554469.003

[B70] Traugott E. C. Dasher R. B. (2001). Regularity in Semantic Change. Cambridge Studies in Linguistics. Cambridge: Cambridge University Press.

[B71] Viola L. (2022). Networks of migrants' narratives: a post-authentic approach to heritage visualisation. J. Comput. Cult. Heritage 2022, 3575863. 10.1145/3575863

[B72] Viola L. (2023). The Humanities in the Digital: Beyond Critical Digital Humanities. London: Palgrave Macmillan.

[B73] Viola L. Fiscarelli M. A. (2021). “From digitised sources to digital data: behind the scenes of (critically) enriching a digital heritage collection,” in Proceedings of the International Conference Collect and Connect: Archives and Collections in a Digital Age, CEUR—Workshops Proceedings, eds A. Weber, M. Heerlien, E. G. Miracle, and K. Wolstencroft, 51–64. Available online at: http://ceur-ws.org/Vol-2810/paper5.pdf (accessed August 16, 2023).

[B74] Viola L. Spence P. J. (2023). Multilingual Digital Humanities. London: Routledge Taylor and Francis Group.

[B75] Xiao Y. Li C. Thürer M. Liu Y. Qu T. (2022). Towards lean automation: fine-grained sentiment analysis for customer value identification. Comput. Indus. Eng. 169, 108186. 10.1016/j.cie.2022.108186

[B76] Zhong N. Ren J. (2022). Using sentiment analysis to study the relationship between subjective expression in financial reports and company performance. Front. Psychol. 13, 949881. 10.3389/fpsyg.2022.949881 35936313 PMC9355555

